# Agreement and Repeatability of Central and Peripheral Refraction by One Novel Multispectral-Based Refractor

**DOI:** 10.3389/fmed.2021.777685

**Published:** 2021-12-09

**Authors:** Weicong Lu, Rongyuan Ji, Wenzhi Ding, Yuyin Tian, Keli Long, Zhen Guo, Lin Leng

**Affiliations:** State Key Laboratory Cultivation Base, Shandong Provincial Key Laboratory of Ophthalmology, Eye Institute of Shandong First Medical University, Qingdao Eye Hospital of Shandong First Medical University, Qingdao, China

**Keywords:** agreement, repeatability, refraction, myopia, ophthalmology

## Abstract

**Purpose:** To evaluate the repeatability of a multispectral-based refractor in central and peripheral refraction measurement, and to assess the agreement of such measurements with objective refraction (OR) and subjective refraction (SR) in patients with myopia.

**Methods:** A total of 60 subjects were recruited in this prospective research. Patients were divided into three groups according to the refractive error. Next, the central and peripheral refraction parameters were measured using multispectral refractive tomography (MRT) before and after cycloplegia. In addition, OR and SR measurements were also performed. The intraobserver repeatability was analyzed using within-subject standard deviation (Sw), test–retest repeatability (TRT), and intraclass correlation coefficient (ICC). Agreement was evaluated using Bland-Altman plot and 95% limits of agreement (LoA).

**Results:** The ICC value of central and peripheral refraction were all higher than 0.97 with or without cycloplegia. The peripheral refraction in the nasal, temporal, superior, and inferior quadrants was slightly worse than other parameters, with the largest error interval being 1.43 D. The 95% LoA of the central refraction and OR or SR ranged from −0.89 to 0.88 D and −1.24 to 1.16 D without cycloplegia, respectively, and from −0.80 to 0.42 D and −1.39 to −0.84 D under cycloplegia, respectively.

**Conclusions:** The novel multispectral refraction topography demonstrated good repeatability in central and peripheral refraction. However, the refraction in the nasal, temporal, superior, and inferior quadrants were not as good as that of central and circle peripheral refraction.

## Introduction

In the past few decades, myopia has emerged as a worldwide public health issue due to its rapidly increasing prevalence (1). The overall prevalence of myopia is ~40% in the United States and >80% in young adults in China (2). In developed countries, 15 to 49% of the adult population suffer from myopia (3). It is worth noting that myopia is a complex disease. Evidence suggests that genetic and environmental factors play important roles in its occurrence and development (4). However, its pathogenesis has not yet been fully elucidated. In recent years, many clinical studies have proposed that visual signals from the peripheral retina might induce myopia (5). Moreover, studies involving animal models have demonstrated that the peripheral retina refraction status dominated refractive changes whenever conflicts occur between the fovea and the peripheral visual signals (6). Therefore, this finding calls for the measurement of both the central and relative peripheral refractive errors with the overarching goal of elucidating the mechanisms of myopic development (7). It has been reported that inhibiting the progression of myopia by reducing the hyperopic defocus of the peripheral retina following a refractive correction is an effective method (8).

A study has revealed that using an autorefractor for objective refraction during the initial process in myopia examination is a reliable method compared with the use of subjective refraction (9). WAM-5500 (Grand Seiko Co., Hiroshima, Japan), a binocular, open-field, infrared, and ref/keratometer, is generally used in the clinic to measure central and peripheral retina refractive because of its well-documented repeatability (10, 11). However, its use is associated with certain difficulties and challenges in patients wearing optic lenses, and the measurement region is limited to a few specific spots only (12). To address these limitations, multispectral refractive tomography (MRT) (version 1.0.5T05C; Thondar, Inc. China), a novel multispectral-based computing system, was designed to measure the spherical equivalent (SE) of a 53-degree fundus field of view within 2–3 s. MRT simultaneously obtains the refractive power of all retinal regions, including the central and peripheral retina, within a certain range. In MRT, there is one internal fixation point rather than having the fixation point being moved in different positions and angles. Given that MRT is a newly introduced device, its repeatability should be investigated to broaden its clinical application. This study aimed to explore the repeatability of the measurements obtained using the MRT device and assess the agreement among the refractive measurements made using MRT, Topcon KR-1 (Topcon, Tokyo, Japan), and subjective refraction.

## Materials and Methods

### Patients

In this prospective study, 60 subjects who visited the Qingdao Eye Hospital of Shandong First Medical University for regular examination in August 2021 were recruited. Only the right eye of each patient was examined in this study. Patients were divided into three groups according to the SE measured by the subjective refraction (NIDEK AOS1500+SSC3): low myopia group (−3.00 D < SE ≤ -0.50 D), moderate myopia group (−6.00 D < SE ≤ -3.00 D), and high myopia group (SE ≤ -6.00 D) (13). The study was approved by the Ethics Committee of Qingdao Eye Hospital of Shandong First Medical University (ChiCTR2100049050) and adhered to the tenets of the Declaration of Helsinki. Signed informed consent was obtained from all patients prior to the conduct of this study.

The enrolled patients met the following inclusion criteria: age > 18 years, astigmatism diopter <3.0 D, no history of cornea refractive surgery, no history of ocular trauma, and agreed to stop wearing contact lenses for at least 2 weeks for soft contact lenses and 4 weeks for rigid gas-permeable contact lenses before the examination.

### Instrument and Methods

**Figure 1:**
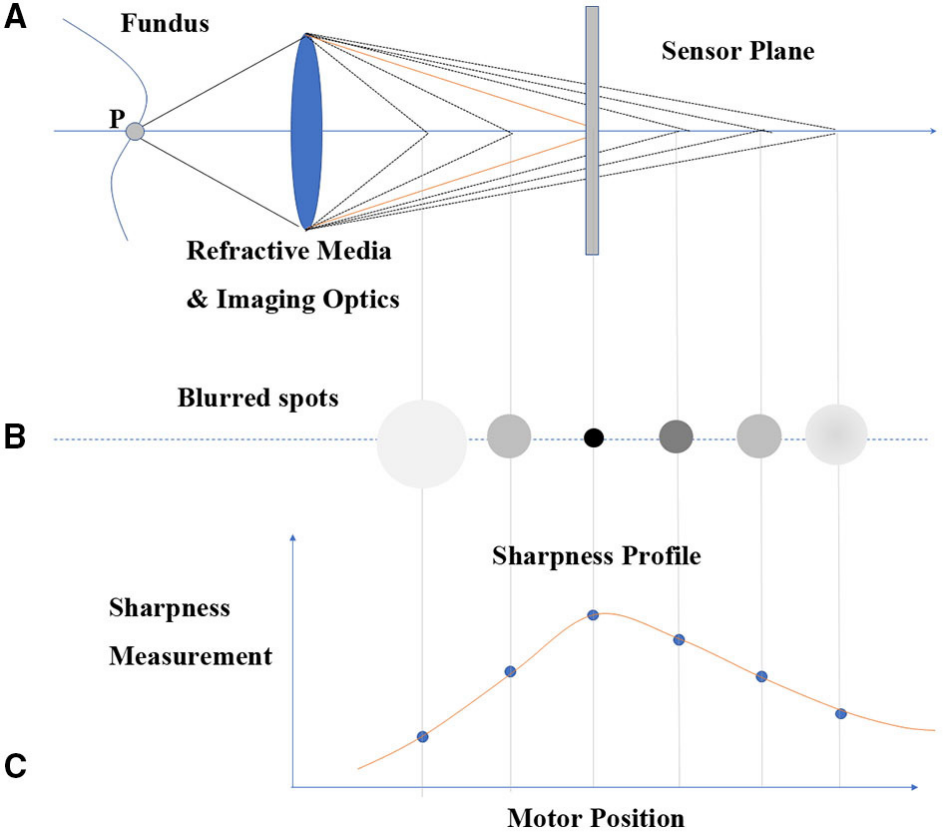
MRT principle diagram. **(A)** Light ray radiation and sensor system. **(B)** Blurred image point with the change of focal length of fundus camera. **(C)** The sharpness profile of changing focal length.

MRT was designed according to the simplified reduced optical model. Light rays were radiated from one ideal point (P) of fundus and were transmitted via an optical path, comprising the refractive media of human eye and imaging optics of fundus camera. Images were ultimately formed on the sensor plane (Figure 1A). When the focal length of the fundus camera was adjusted, the image point was accommodated accordingly into relative defocused status, shaping into a blurred spot with different sizes and gray levels (Figure 1B). In the fundus camera imaging process, the radiation originated from a fundus surface rather than from an ideal point. Consequently, the fundus image was blurred to different degrees in different focal lengths, which was controlled by the focus motor. A focus measure, such as the Sobel operator, was applied to measure the sharpness of each region of interest (ROI) in the fundus, representing a particular viewing angle such as the central view, nasal 10° eccentricity, and temporal 25°. As shown in Figure 1C, a sharpness profile of the changes in focal length was obtained for a particular viewing angle. The maximum sharpness of the sharpness profile correlated with the situation when point P was focused on the sensor plane. The motor position when the maximum sharpness was obtained in a given imaging optical system represents the specific optic setup from which the human eye refraction was calculated. A sequential calculation for all ROIs in the defined field of view generated a refraction tomography. Notably, the device can provide both central and peripheral refractive errors with different eccentricities.

**Figure 2:**
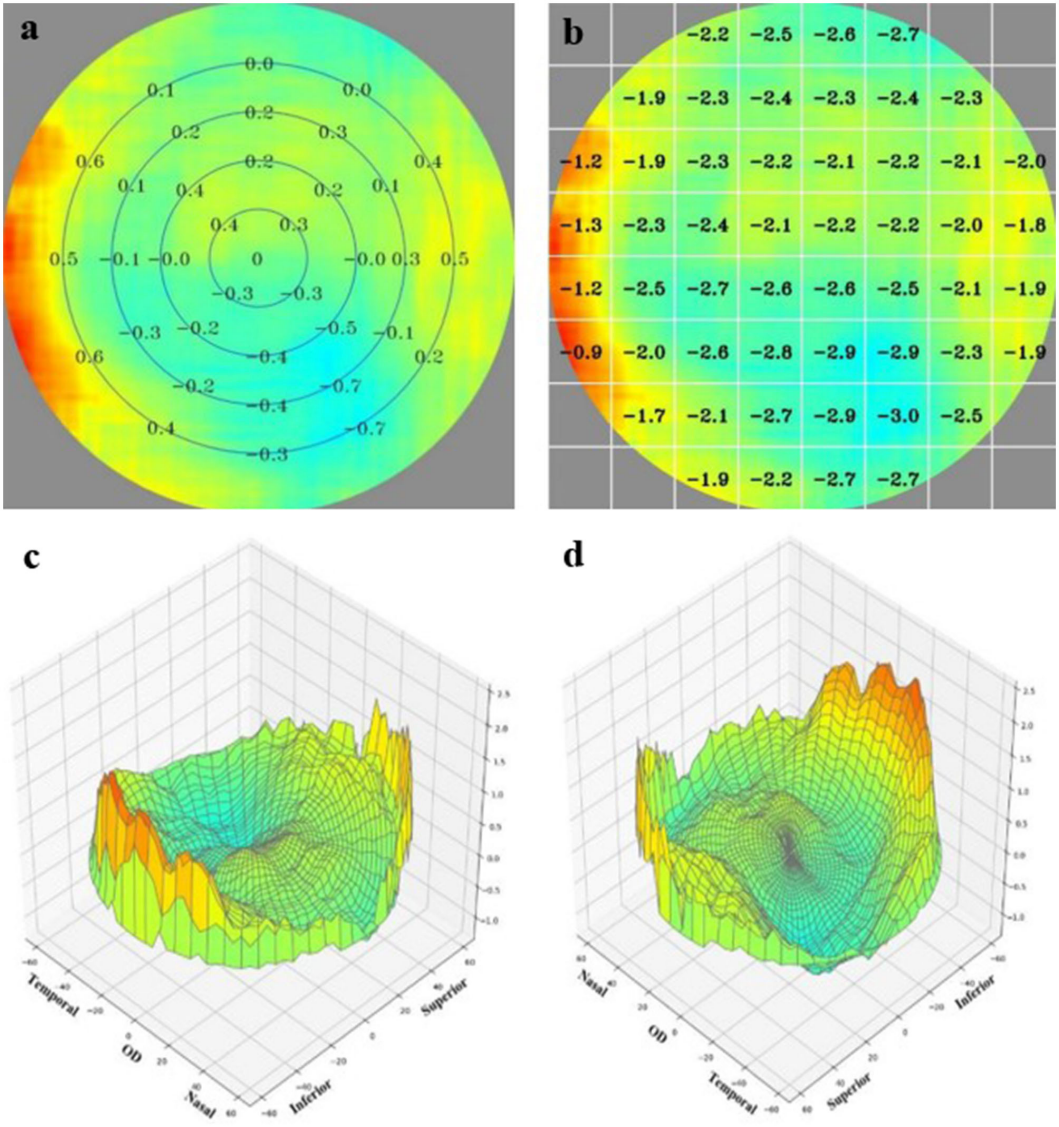
The result provided by the MRT analysis system. **(a)** The relative peripheral refraction defocus value. **(b)** The block-refraction of absolute refraction value. **(c,d)** A direct view of the relative fraction status of the retinal by three-dimensional images viewing from superotemporal and inferonasal, respectively.

Next, image analysis was performed and an algorithm was used to decouple and generate the refractive value of each imaging data point. This approach could determine the SE of 128 × 128 points on a 53-degree field of view of the fundus, with a data point of 0.5° in between. After each data point was acquired, a set of images processed by a custom compensation software was obtained using the color-coded approach (Figure 2). Relative peripheral refraction defocus was the difference between the absolute refraction and the central macular refraction and was translated into color images (Figure 2a). Block-refraction provided an absolute refraction value using each value as mean data for each corresponding block (Figure 2b). The three-dimensional (3D) images of superotemporal and inferonasal retina provide a direct view of the relative fraction status of the retina (Figures 2c,d).

Prior to the experiment, the patients were subjected to routine examinations, including visual acuity examination, slit-lamp examination of the anterior segment, and fundus evaluations. The examinations were conducted between 1,000 and 1,700 h by an experienced doctor to avoid the influence of diurnal variation (14). Initially, patients were positioned on the headrest and asked to fix their attention to the internal target. Next, the patients were asked to blink twice before measurement to ensure that the tear film coating cornea surface was intact. All MRT measurements were carried out by an experienced doctor. Each patient was examined three times to evaluate the intraobserver repeatability. Objective refraction (OR) using Topcon KR-1 and subjective refraction (SR) were conducted by another doctor who was blinded to the previous examination results. Next, compound tropicamide 0.5% and phenylephrine 0.5% (SINQI Pharmaceutical Co., Ltd., Shenyang, China) was used three times, with an interval of 5 min to induce cycloplegia until the pupil diameter reached 7–8 mm to relax the accommodation. The MRT, OR, and SR examinations were repeated by the same doctor to minimize the operator-related error.

The parameters obtained using MRT for further analysis were as follows: central refractive error (CRE); total refraction difference value, which indicates the average peripheral refractive error from the center to the peripheral 53° of the retina (TRDV); refraction difference value-15 which indicates the average paracentral refractive error from the center to 15° of the retina (RDV-15); refraction difference value-30 and 45 (RDV-30, and RDV-45, respectively) which indicate the average peripheral refractive error at 30 and 45° of the posterior retina, respectively, refraction difference value-inferior (RDV-I); refraction difference value-superior (RDV-S); refraction difference value-nasal (RDV-N); and refraction difference value-temporal (RDV-T). The measurement quality was estimated by a computer to avoid the influence of iris reflection, eye blinking, and dim illumination, and only those results with a quality score of >80% were recorded for further analysis. For the SR examination, the OR was used as the baseline value instead of the MRT so as not to influence the examination being conducted.

### Statistical Analysis

All statistical analyses were performed using SPSS software (version 24.0; IBM Corporation, Armonk, NY) and Medcalc software (version 24.0; IBM Corporation, Armonk, NY). All data were recorded as mean ± standard deviation (SD). Data distribution was analyzed by the Kolmogorov–Smirnov test to determine normally distributed data (*P* > 0.05). To assess the intraoperator repeatability of MRT, one-way analysis of variance (ANOVA) was used to calculate the within-subject standard deviation (Sw), the test–retest repeatability (TRT), and the intraclass correlation coefficient (ICC). The Sw is the intraoperator deviation derived from the three consecutive measurements. When the TRT is equal to 2.77 Sw represents the 95% measurement deviation interval within which the measurement error should lie. ICC is a common parameter used to evaluate repeatability and is defined as the ratio of variance between individual measurements to the sum. In clinical application, an ICC value larger than 0.9 indicates high repeatability. However, an ICC value of 0.75 is acceptable in statistical applications (15).

The mean of the three consecutive measurements was used in assessing agreement with the SR and OR. For the agreement evaluation, the MedCalc statistical software (version 18.2.1, Ostend, Belgium) was used to draw the Bland-Altman plots. The 95% limit of agreement (LoA) was drawn according to the mean difference ± 1.96 SD between two methods, and it indicates the measurement error of these methods (16).

## Results

Sixty patients were recruited in this study, and the average age was 27.25 ± 6.70 years (range: 18–36 years). The mean SE before and after cycloplegia was −5.28 ± 1.95 D and −4.93 ± 1.94 D for OR, respectively, and −5.32 ± 1.82 D and 5.01 ± 1.85 D for SR, respectively. The mean SE in each group with sample sizes of 19, 21, and 20 was −2.51 ± 0.82 D, −5.02 ± 0.61 D, and −7.13 ± 0.87 D, respectively.

### Intraoperator Repeatability

Table 1 shows the repeatability of MRT in central and peripheral refraction measurements in patients with myopia before cycloplegia. The ICC values were all above 0.97. Similarly, the Sw and TRT results supported the good repeatability of CRE, TRDV, RDV-15, RDV-30, and RDV-45, but the RDV of different quadrants was slightly worse. The largest error interval was 1.43 D, indicating that the variation among the measurements for superior peripheral refraction could reach 1.43 D. However, the repeatability of these parameters significantly improved after cycloplegia, with all ICC values higher than 0.99. The Sw and TRT were smaller in CRE, TRDV, RDV-15, RDV-30, and RDV-45 in the cycloplegia group than in the non-cycloplegia group (Table 2). Notably, the RDV for the different quadrants remained the same in the cycloplegia group compared with that in the non-cycloplegia group, with the exception of RDV-I measurement, as its repeatability improved.

**Table 1:** Intraobserver repeatability outcomes of central and peripheral refraction using MRT without cycloplegia.

**Parameters**	**Mean**	**SD**	**Sw**	**TRT**	**ICC**
CRE	−5.28	1.96	0.37	1.04	0.988
TRDV	−4.53	1.89	0.36	1.02	0.987
RDV-15	−5.09	1.89	0.42	1.16	0.983
RDV-30	−4.77	1.88	0.38	1.06	0.986
RDV-45	−4.55	1.88	0.35	0.98	0.988
RDV-S	−4.80	1.88	0.51	1.43	0.974
RDV-I	−4.41	1.94	0.50	1.39	0.977
RDV-T	−4.85	2.04	0.43	1.20	0.984
RDV-N	−4.07	1.94	0.51	1.42	0.976

*CRE, central refractive error; TRDV, peripheral refractive error from center to peripheral 53°of retina; RDV-15, the difference of CRE and paracentral refractive error from center to 15°of retina; RDV-30, the difference of CRE and paracentral refractive error from center to 30°of retina; RDV-45, the difference of CRE and paracentral refractive error from center to 45°of retina; RDV-S, refraction difference value-superior; RDV-I, refraction difference value-inferior; RDV-T, refraction difference value- refraction difference value-temporal; RDV-N, refraction difference value-nasal; SD, standard deviation; Sw, standard deviation; TRT, test–retest repeatability; ICC, intraclass correlation coefficient*.

**Table 2:** Intraobserver repeatability outcomes of central and peripheral refraction using MRT with cycloplegia.

**Parameters**	**Mean**	**SD**	**Sw**	**TRT**	**ICC**
CRE	−4.74	1.89	0.27	0.76	0.993
TRDV	−3.56	1.82	0.27	0.75	0.992
RDV-15	−4.32	1.90	0.33	0.92	0.990
RDV-30	−3.95	1.86	0.30	0.852	0.991
RDV-45	−3.63	1.85	0.28	0.79	0.992
RDV-S	−3.71	1.90	0.45	1.25	0.980
RDV-I	−3.49	1.90	0.37	1.03	0.987
RDV-T	−3.87	1.95	0.50	1.39	0.978
RDV-N	−3.09	1.98	0.50	1.41	0.977

*CRE, central refractive error; TRDV, peripheral refractive error from center to peripheral 53°of retina; RDV-15, the difference of CRE and paracentral refractive error from center to 15°of retina; RDV-30, the difference of CRE and paracentral refractive error from center to 30°of retina; RDV-45, the difference of CRE and paracentral refractive error from center to 45°of retina; RDV-S, refraction difference value-superior; RDV-I, refraction difference value-inferior; RDV-T, refraction difference value- refraction difference value-temporal; RDV-N, refraction difference value-nasal; SD, standard deviation; Sw, standard deviation; TRT, test–retest repeatability; ICC, intraclass correlation coefficient*.

Furthermore, we analyzed the repeatability of different refractive errors. As shown in Tables 3–5, the CRE, TRDV, RDV-15, RDV-30, and RDV-45 all showed good repeatability, and the high myopia group without cycloplegia showed the highest degree of repeatability. Moreover, the RDV-I, RDV-S, RDV-N, and RDV-T demonstrated a lower degree of repeatability than the CRE, TRDV, RDV-15, RDV-30, and RDV-45, and patients in the low myopia group were the most easily influenced by these parameters. The ICC values for the RDV-I, RDV-S, RDV-N, and RDV-T in the low myopia group ranged from 0.83 to 0.89, and increased with the increment of myopia diopter. Tables 6–8 show the results obtained after cycloplegia. Moreover, the repeatability of the measurements in the quadrants were slightly of lower degree than that of CRE, TRDV, RDV-15, RDV-30, and RDV-45. However, all ICC values were higher than 0.9, indicating that repeatability remained good for the three groups.

**Table 3:** Intraobserver repeatability outcomes of central and peripheral refraction using MRT without cycloplegia of low myopia group.

**Parameters**	**Mean**	**SD**	**Sw**	**TRT**	**ICC**
CRE	−2.97	0.82	0.41	1.13	0.909
TRDV	−2.45	0.84	0.38	1.07	0.917
RDV-15	−3.03	1.01	0.42	1.17	0.937
RDV-30	−2.71	0.95	0.38	1.06	0.942
RDV-45	−2.47	0.86	0.36	1.01	0.934
RDV-S	−2.75	0.84	0.52	1.44	0.833
RDV-I	−2.43	1.19	0.63	1.76	0.889
RDV-T	−2.65	0.92	0.48	1.34	0.896
RDV-N	−1.98	1.08	0.62	1.71	0.869

*CRE, central refractive error; TRDV, peripheral refractive error from center to peripheral 53°of retina; RDV-15, the difference of CRE and paracentral refractive error from center to 15°of retina; RDV-30, the difference of CRE and paracentral refractive error from center to 30°of retina; RDV-45, the difference of CRE and paracentral refractive error from center to 45°of retina; RDV-S, refraction difference value-superior; RDV-I, refraction difference value-inferior; RDV-T, refraction difference value- refraction difference value-temporal; RDV-N, refraction difference value-nasal; SD, standard deviation; Sw, standard deviation; TRT, test–retest repeatability; ICC, intraclass correlation coefficient*.

**Table 4:** Intraobserver repeatability outcomes of central and peripheral refraction using MRT without cycloplegia of moderate myopia group.

**Parameters**	**Mean**	**SD**	**Sw**	**TRT**	**ICC**
CRE	−5.41	0.95	0.39	1.09	0.936
TRDV	−4.76	1.09	0.39	1.09	0.955
RDV-15	−5.31	1.05	0.50	1.39	0.912
RDV-30	−5.00	1.05	0.44	1.24	0.933
RDV-45	−4.78	1.07	0.39	1.09	0.953
RDV-S	−5.05	1.11	0.57	1.59	0.897
RDV-I	−4.56	1.23	0.46	1.27	0.949
RDV-T	−5.12	1.33	0.42	1.16	0.964
RDV-N	−4.33	1.09	0.53	1.47	0.920

*CRE, central refractive error; TRDV, peripheral refractive error from center to peripheral 53°of retina; RDV-15, the difference of CRE and paracentral refractive error from center to 15°of retina; RDV-30, the difference of CRE and paracentral refractive error from center to 30°of retina; RDV-45, the difference of CRE and paracentral refractive error from center to 45°of retina; RDV-S, refraction difference value-superior; RDV-I, refraction difference value-inferior; RDV-T, refraction difference value- refraction difference value-temporal; RDV-N, refraction difference value-nasal; SD, standard deviation; Sw, standard deviation; TRT, test–retest repeatability; ICC, intraclass correlation coefficient*.

**Table 5:** Intraobserver repeatability outcomes of central and peripheral refraction using MRT without cycloplegia of high myopia group.

**Parameters**	**Mean**	**SD**	**Sw**	**TRT**	**ICC**
CRE	−7.33	0.85	0.32	0.89	0.954
TRDV	−6.39	0.81	0.35	0.98	0.931
RDV-15	−6.97	0.78	0.30	0.85	0.948
RDV-30	−6.63	0.76	0.30	0.84	0.945
RDV-45	−6.43	0.78	0.33	0.92	0.936
RDV-S	−6.60	0.92	0.46	1.29	0.902
RDV-I	−6.26	0.90	0.41	1.15	0.920
RDV-T	−6.79	0.94	0.46	1.29	0.904
RDV-N	−5.92	0.90	0.38	1.05	0.936

*CRE, central refractive error; TRDV, peripheral refractive error from center to peripheral 53°of retina; RDV-15, the difference of CRE and paracentral refractive error from center to 15°of retina; RDV-30, the difference of CRE and paracentral refractive error from center to 30°of retina; RDV-45, the difference of CRE and paracentral refractive error from center to 45°of retina; RDV-S, refraction difference value-superior; RDV-I, refraction difference value-inferior; RDV-T, refraction difference value- refraction difference value-temporal; RDV-N, refraction difference value-nasal; SD, standard deviation; Sw, standard deviation; TRT, test–retest repeatability; ICC, intraclass correlation coefficient*.

**Table 6:** Intraobserver repeatability outcomes of central and peripheral refraction using MRT with cycloplegia of low myopia group.

**Parameters**	**Mean**	**SD**	**Sw**	**TRT**	**ICC**
CRE	−2.46	0.54	0.24	0.67	0.921
TRDV	−1.45	0.65	0.20	0.57	0.965
RDV-15	−2.03	0.63	0.22	0.63	0.952
RDV-30	−1.71	0.63	0.19	0.54	0.966
RDV-45	−1.44	0.65	0.21	0.60	0.962
RDV-S	−1.68	0.83	0.36	1.02	0.925
RDV-I	−1.31	0.77	0.29	0.81	0.951
RDV-T	−1.65	0.73	0.23	0.65	0.964
RDV-N	−0.92	0.97	0.35	0.97	0.952

*CRE, central refractive error; TRDV, peripheral refractive error from center to peripheral 53°of retina; RDV-15, the difference of CRE and paracentral refractive error from center to 15°of retina; RDV-30, the difference of CRE and paracentral refractive error from center to 30°of retina; RDV-45, the difference of CRE and paracentral refractive error from center to 45°of retina; RDV-S, refraction difference value-superior; RDV-I, refraction difference value-inferior; RDV-T, refraction difference value- refraction difference value-temporal; RDV-N, refraction difference value-nasal; SD, standard deviation; Sw, standard deviation; TRT, test–retest repeatability; ICC, intraclass correlation coefficient*.

**Table 7:** Intraobserver repeatability outcomes of central and peripheral refraction using MRT with cycloplegia of moderate myopia group.

**Parameters**	**Mean**	**SD**	**Sw**	**TRT**	**ICC**
CRE	−4.86	0.81	0.31	0.88	0.943
TRDV	−3.74	1.00	0.28	0.78	0.972
RDV-15	−4.50	0.88	0.36	1.00	0.939
RDV-30	−4.13	0.88	0.32	0.88	0.954
RDV-45	−3.82	0.95	0.28	0.79	0.968
RDV-S	−3.83	1.31	0.54	1.51	0.934
RDV-I	−3.72	1.04	0.37	1.04	0.953
RDV-T	−4.17	1.28	0.67	1.86	0.895
RDV-N	−3.21	1.12	0.56	1.56	0.899

*CRE, central refractive error; TRDV, peripheral refractive error from center to peripheral 53°of retina; RDV-15, the difference of CRE and paracentral refractive error from center to 15°of retina; RDV-30, the difference of CRE and paracentral refractive error from center to 30°of retina; RDV-45, the difference of CRE and paracentral refractive error from center to 45°of retina; RDV-S, refraction difference value-superior; RDV-I, refraction difference value-inferior; RDV-T, refraction difference value- refraction difference value-temporal; RDV-N, refraction difference value-nasal; SD, standard deviation; Sw, standard deviation; TRT, test–retest repeatability; ICC, intraclass correlation coefficient*.

**Table 8:** Intraobserver repeatability outcomes of central and peripheral refraction using MRT with cycloplegia of high myopia group.

**Parameters**	**Mean**	**SD**	**Sw**	**TRT**	**ICC**
CRE	−6.77	0.86	0.25	0.71	0.968
TRDV	−5.39	0.94	0.31	0.86	0.961
RDV-15	−6.30	0.83	0.38	1.05	0.919
RDV-30	−5.88	0.85	0.37	1.03	0.926
RDV-45	−5.52	0.90	0.33	0.93	0.949
RDV-S	−5.52	1.09	0.41	1.13	0.948
RDV-I	−5.32	1.09	0.43	1.19	0.944
RDV-T	−5.66	1.10	0.47	1.32	0.933
RDV-N	−5.01	1.16	0.56	1.57	0.905

*CRE, central refractive error; TRDV, peripheral refractive error from center to peripheral 53°of retina; RDV-15, the difference of CRE and paracentral refractive error from center to 15°of retina; RDV-30, the difference of CRE and paracentral refractive error from center to 30°of retina; RDV-45, the difference of CRE and paracentral refractive error from center to 45°of retina; RDV-S, refraction difference value-superior; RDV-I, refraction difference value-inferior; RDV-T, refraction difference value- refraction difference value-temporal; RDV-N, refraction difference value-nasal; SD, standard deviation; Sw, standard deviation; TRT, test–retest repeatability; ICC, intraclass correlation coefficient*.

### Agreement

**Figure 3:**
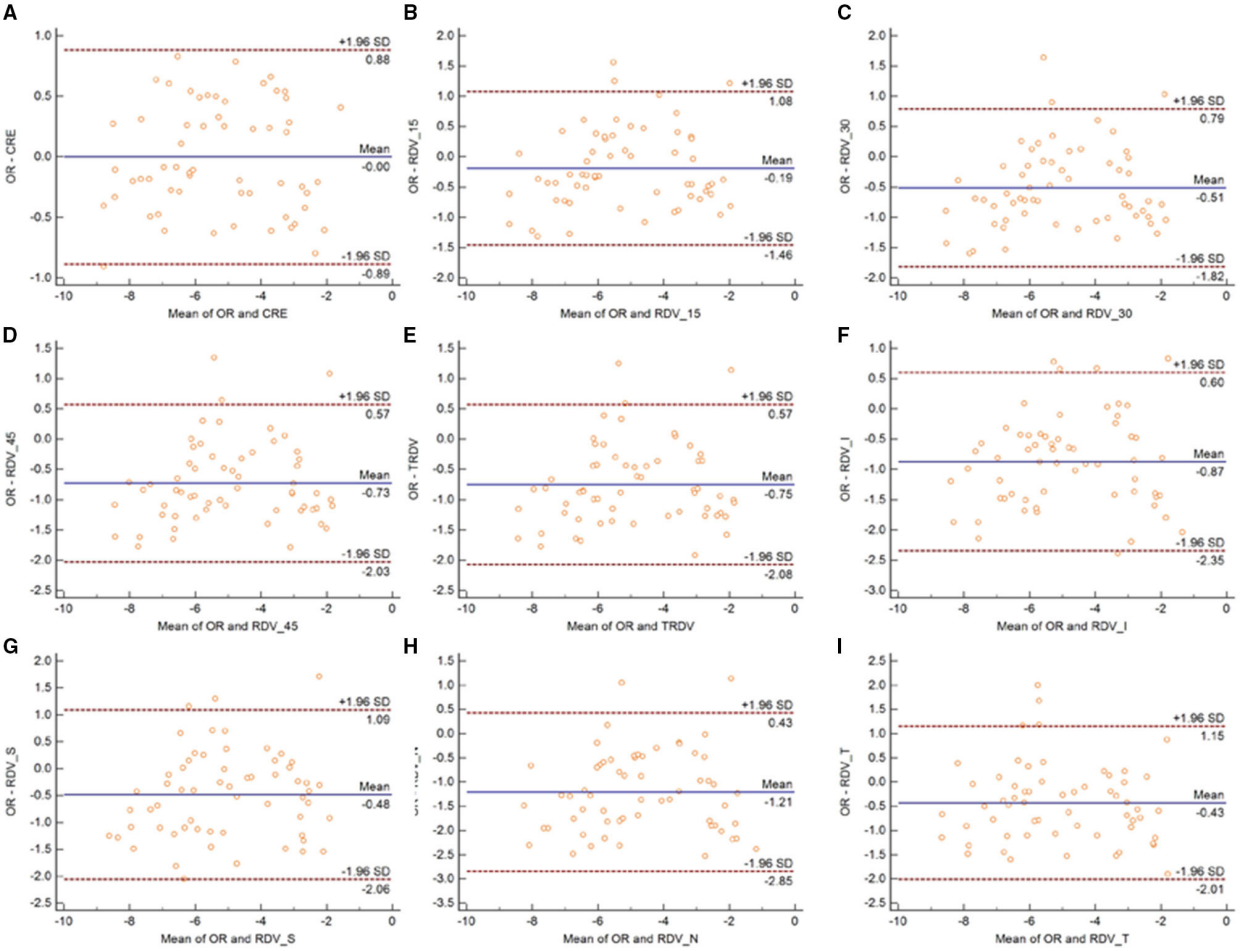
Bland–Altman plots between SR and CRE **(A)**, RDV-15 **(B)**, RDV-30 **(C)**, RDV-45 **(D)**, TRVD **(E)**, RDV-I **(F)**, RDV-S **(G)**, RDV-N **(H)**, and RDV-T **(I)** in non-cycloplegia eyes. The solid line shows the mean difference (bias), and the upper and lower lines represent 95% limits of agreement.

**Figure 4:**
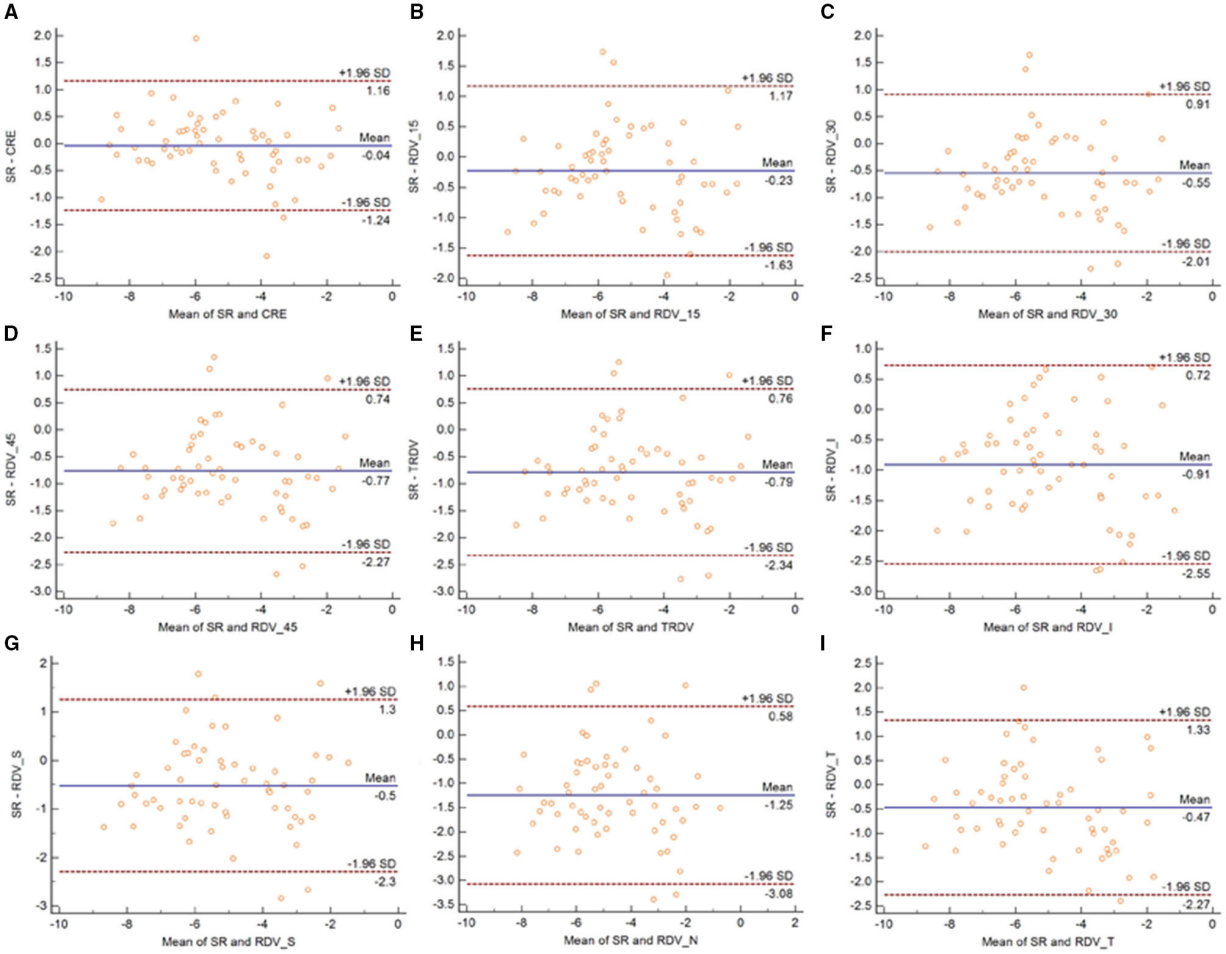
Bland–Altman plots between OR and CRE **(A)**, RDV-15 **(B)**, RDV-30 **(C)**, RDV-45 **(D)**, TRVD I **(E)**, RDV-I **(F)**, RDV-S **(G)**, RDV-N **(H)**, and RDV-T **(I)** in non-cycloplegia eyes. The solid line shows the mean difference (bias), and the upper and lower lines represent 95% limits of agreement.

Figure 3 shows the Bland-Altman plots comparing MRT and OR before cycloplegia. Results demonstrated that there was no significant difference in CRE and OR, and the 95% LoA ranged from −0.89 to 0.88 D, indicating a good agreement. However, the difference between peripheral refraction and OR is higher compared with the difference between CRE and OR. In addition, the refractive error in the peripheral retina was smaller than the OR, although the interval of 95% LoA was stable (nearly 2.5 D). Meanwhile, the RDV-I, RDV-S, RDV-N, and RDV-T values increased up to nearly 3.0 D. A similar result as regard the agreement between MRT and SR was observed (Figure 4). The 95% LoA of CRE and SR ranged from −1.24 to 1.14 D, and RDV-I, RDV-S, RDV-N, and RDV-T demonstrated a lower degree of agreement.

**Figure 5:**
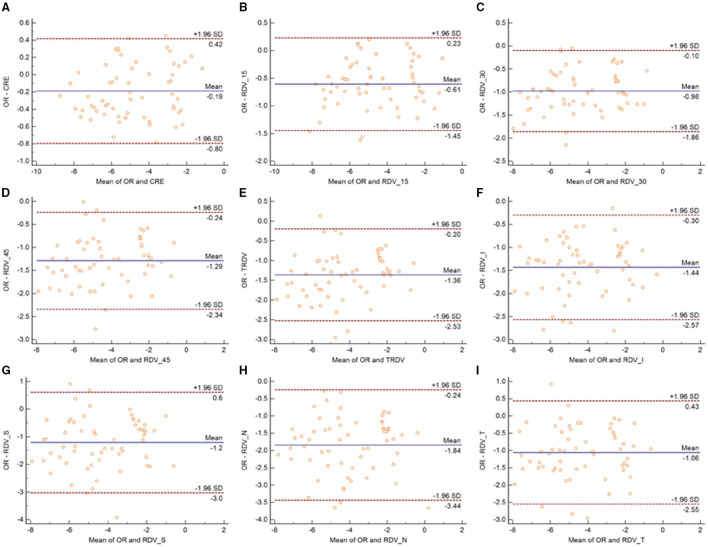
Bland–Altman plots between SR and CRE **(A)**, RDV-15 **(B)**, RDV-30 **(C)**, RDV-45 **(D)**, TRVD **(E)**, RDV-I **(F)**, RDV-S **(G)**, RDV-N **(H)**, and RDV-T **(I)** in cycloplegia eyes. The solid line shows the mean difference (bias), and the upper and lower lines represent 95% limits of agreement.

**Figure 6:**
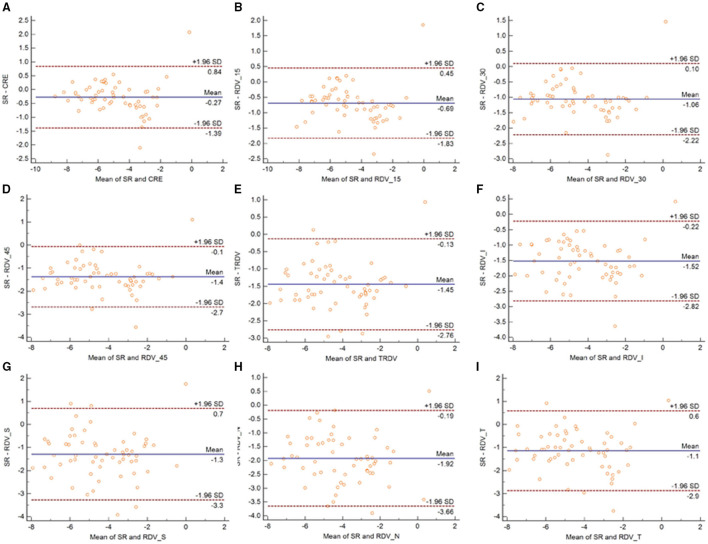
Bland–Altman plots between OR and CRE **(A)**, RDV-15 **(B)**, RDV-30 **(C)**, RDV-45 **(D)**, TRVD **(E)**, RDV-I **(F)**, RDV-S **(G)**, RDV-N **(H)**, and RDV-T **(I)** in cycloplegia eyes. The solid line shows the mean difference (bias), and the upper and lower lines represent 95% limits of agreement.

Similarly, the 95% LoA of CRE and OR ranged from −0.80 to 0.42 D after cycloplegia, suggesting that cycloplegia could enhance the agreement given that the accommodation was relaxed (Figure 5). Notably, the peripheral refraction was more remarkable in RDV-45. RDV-S demonstrated the largest 95% LoA, which ranged from −3.0 to 0.6 D, indicating a low degree of agreement in different quadrants. Moreover, RDV-S showed a higher degree of the agreement with the OR group than with the SR group, and it had the largest interval that ranged from −3.3 to 0.7 D (Figure 6).

## Discussion

Myopia, a multi-factor-related disease, is the most prevalent disorder worldwide (17). Previous studies have confirmed that peripheral hyperopia refractive status plays a crucial role in myopia progression, especially in patients who need spectacle correction (18). Therefore, measurement of the peripheral refractive error is an important aspect in clinical application (19). Although the measurement can be done using the WAM-5500, the intrinsic limitations of spots calculation restrict its further application (11). Such disadvantages can be overcome by MRT, a novel device that can measure the large areas of peripheral refraction. This study explored the repeatability of using MRT to measure central and peripheral refraction before and after cycloplegia in different groups. The results were also compared with the OR and SR measurements obtained under the same conditions.

The current results demonstrated that MRT could provide reproducible results for CRE, TRDV, RDV-15, RDV-30, and RDV-45 without cycloplegia, and the RDV showed a slightly lower degree of repeatability in the four different quadrants. It should be noted that MRT adopts a mechanism similar to that of autorefractors. To our best knowledge, this study was the first to evaluate the repeatability of MRT. Our findings were consistent with that of other studies that evaluated the repeatability of autorefractors in non-cycloplegia refractive error. For example, Nguyen and Berntsen (10) found that the sphere was −0.34 D, with a 95% LoA ranging from −0.37 to 0.32 D without cycloplegia. Allen et al. (20) reported that the repeatability of an autorefractor had a 95% LoA ranging from −0.45 to 0.47 D. Moreover, Elliott et al. (21) investigated the repeatability of Nikon NRK-8000, Nidek AR-1000, and SR, and they have found that the COR values for these three methods were 0.71, 0.26, and 0.61 D, respectively. The above findings are consistent with our results, confirming that MRT could demonstrate a good repeatability in central refraction measurement. In addition, the repeatability of MRT was significantly enhanced after the cycloplegia, which may be attributed to the fact that the cycloplegia could have relaxed the ciliary muscle, thereby reducing the accommodation reflex. Nguyen and Berntsen (10) found that when the pupil size was 6 mm, the repeatability of sphere diopter was ± 0.32 D. Hernandez-Moreno et al. (22) also investigated the repeatability of SE in a pediatric population, and they found that a 95% LoA ranging from −0.66 to +0.65 D with cycloplegia and from −1.38 to +1.74 D without cycloplegia. These findings suggested that the autorefractor had a low repeatability under non-cycloplegia conditions, but such a degree of repeatability is acceptable under cycloplegic conditions. Their results are consistent with our finding showing that cycloplegia could enhance the repeatability of MRT.

The peripheral refraction error determined using MRT also showed that the repeatability of the TRDV, RDV-15, RDV-30, and RDV-45 was better than that of the peripheral refractive error measured in the quadrants. RDV-S showed the lowest degree of repeatability in the non-cycloplegia group, but its repeatability was better under cycloplegic conditions. Notably, the RDV-N was the least easily influenced. We hypothesize that the upper eyelid pressure plays a role in peripheral refraction. As previously reported, eyelid pressure could induce corneal topographic changes and eyelid morphometry, so peripheral refraction would also be influenced by corneal topographic changes (23). Collectively, these results confirmed that the repeatability of MRT for the peripheral refraction measurements was associated with the measurement regions. The asphericity of the retina (especially in myopic eyes) may cause differences in the intensity of light ray distribution on the retina in different regions. This may explain the worse measurement results of the quadrants than the data of the circle. WAM-5500, frequently used in peripheral autorefraction, was selected as the reference since no research about the novel MRT has been published (11). In an investigation by Moore and Berntsen (11), the repeatability of cycloplegia autorefraction in normal eyes was ± 0.21 D, ± 0.42 D, ± 0.60 D, ± 0.73 D, ± 0.36 D, ± 0.47 D, and ± 0.88 D for central, 20°, 30°, 40° nasal, 20°, 30°, and 40°temporal, respectively, which are consistent with our results. By contrast, we found that the repeatability did not decrease as eccentricity increased. This phenomenon may be attributed to the fact that the spot measurement made by the WAM 5500, which has an open-field design, was based on the fixation point the patient stared at. When autorefractor measurements are being obtained at higher eccentricities, any changes in fixation could induce a measurement error and a lateral pupil misalignment, which also influence repeatability. Fedtke et al. (24) reported that even a 0.27 mm lateral misalignment of the pupil center would cause a 0.25 D change in peripheral defocus when measuring at 30° in the periphery of a myopic eye. Meanwhile, the MRT could calculate the entire retinal refraction in one measurement and analyze the peripheral refraction through adjustment in the focal length of the fundus camera in order to eliminate influence of misalignment of the pupil center.

This study found that the agreement of MRT with the OR or SR decreased as eccentricity increased. The majority of the patients had a noticeable peripheral hyperopia, consistent with previous findings (24). However, the average difference in the peripheral refractive error between MRT and OR was smaller than that between MRT and SR, and the central refractive error in OR was better than that in SR. This may be due to the fact that the SR could measure the exact macular refractive error and could consider the posterior retina as a sphere although its actual shape is ellipse. Liao et al. (25) have reported that the 95% LoA between MRT and autorefractometer ranged from −1.43 to 1.83 D. Similarly, we found that the same conclusion and the 95% interval of LoA was narrower. The MRT measurement in the peripheral retina may increase the difference compared with central retina, but it would still maintain the reliability. Carracedo et al. (26) compared two wavefront autorefractors (Eye Refract and VX110), and discovered the 95% LoA of refraction ranged from −0.99 to 0.59 D. We obtained the same conclusion: MRT is a good technique for central refractive error measurement, even under induced cycloplegia. The results showed that the cycloplegia group had significant peripheral hyperopia compared with the non-cycloplegia group, indicating that the patients could have more peripheral hyperopia status (0.5 D). This finding suggested that care should be taken during peripheral refractive measurement in patients with cycloplegia.

Although the repeatability of all patients with or without cycloplegia showed acceptable repeatability in central and peripheral refraction, we further investigated the relationship between repeatability and the degree of myopia. It was found that the repeatability in the quadrants was worse than others in all three groups, and cycloplegia could improve repeatability. We speculate that cycloplegia improved the repeatability because it enlarged the pupil by nearly 6.0 mm, which is significantly larger than the pupil under natural conditions. Therefore, peripheral measurements of a large pupil size could be easily conducted, and the lens was relaxed under cycloplegia, ensuring constant peripheral lens refraction.

One limitation of this study was that we only evaluated the intraoperator repeatability, whereas repeatability in different time points was not evaluated. In addition, the patients were asked to remove their glasses during measurement. However, as is well-known, peripheral hyperopia inducing myopia mostly happens in patients wearing glasses or lenses. Therefore, future studies should evaluate patients with glasses and lenses. In this study, we only assessed the repeatability of MRT without comparing it with other peripheral wavefront autorefractors. A gold standard in measuring peripheral refraction remains inexistent. Future studies should compare MRT with other devices to gain insights on the introduction of MRT in clinical applications.

## Conclusion

The novel MRT demonstrated good repeatability in central and peripheral refraction measurements. However, the repeatability of the measurements in the nasal, temporal, superior, and inferior quadrants were not as good as that in the central and circle peripheral refractions. Furthermore, we found that cycloplegia relaxed the accommodation and thus could improve repeatability.

## Data Availability Statement

The raw data supporting the conclusions of this article will be made available by the authors, without undue reservation.

## Ethics Statement

The studies involving human participants were reviewed and approved by the Ethics Committee of Qingdao Eye Hospital of Shandong First Medical University. The patients/participants provided their written informed consent to participate in this study. Written informed consent was obtained from the individual(s) for the publication of any potentially identifiable images or data included in this article.

## Author Contributions

All authors listed have made a substantial, direct, and intellectual contribution to the work and approved it for publication.

## Funding

This work was supported by the Medical and Health Development Grant of Shandong Province, China (202007020431).

## Conflict of Interest

The authors declare that the research was conducted in the absence of any commercial or financial relationships that could be construed as a potential conflict of interest.

## Publisher's Note

All claims expressed in this article are solely those of the authors and do not necessarily represent those of their affiliated organizations, or those of the publisher, the editors and the reviewers. Any product that may be evaluated in this article, or claim that may be made by its manufacturer, is not guaranteed or endorsed by the publisher.
